# An Uncommon Case of Secondary Organizing Pneumonia Due to Influenza A Infection

**DOI:** 10.7759/cureus.77043

**Published:** 2025-01-06

**Authors:** Nicholsan Jesiah, Yathukulan Siva, Pakkiyaretnam Mayurathan

**Affiliations:** 1 University Medical Unit, Teaching Hospital - Batticaloa, Batticaloa, LKA; 2 Department of Clinical Sciences, Faculty of Healthcare Sciences, Eastern University of Sri Lanka, Batticaloa, LKA

**Keywords:** high resolution computed tomography of chest, influenza a, methyleprednisolone, secondary organizing pneumonia, viral pneumonia

## Abstract

Secondary organizing pneumonia (SOP) is defined as a lung disease process caused by pulmonary tissue injury. SOP may rarely occur after influenza A infections, including H1N1 influenza A. We present a case of a 62-year-old woman who was diagnosed with viral pneumonia caused by type A influenza, which was confirmed by nasopharyngeal swab reverse transcription-polymerase chain reaction (RT-PCR). Her abnormal chest shadows and oxygen demand did not improve despite antiviral therapy. Given her clinical deterioration, a high-resolution CT of the chest (HRCT-chest) was done, which confirmed SOP. She was managed with methylprednisolone pulse therapy followed by a tapering regime in addition to standard treatment. This report underscores the need for physicians to remain vigilant about the possibility of OP even in cases of viral pneumonia.

## Introduction

The flu virus is a frequent cause of acute respiratory infections during worldwide outbreaks. Organizing pneumonia (OP) is a clinical syndrome characterized by systemic symptoms with alveolar consolidations on imaging with restrictive type lung function test and airways with abnormal granulation tissues [1]. It consists of two major types: cryptogenic organizing pneumonia (COP), if no known cause is identified, and secondary organizing pneumonia (SOP) [2]. Common symptoms of OP include fever, lung symptoms such as exertional dyspnea, resting dyspnea, and a persistent non-productive cough, and constitutional symptoms such as malaise, weight loss, and loss of appetite [3].

Causes of SOP include viral infections such as coronavirus disease 2019 (COVID-19) and bacterial, fungal, and autoimmune causes; it can also be drug-induced. One such well-documented virus is influenza type A [4]. The diagnosis of OP can be confirmed through a transbronchial lung biopsy (TBLB) and analysis of bronchoalveolar lavage fluid (BALF). A key indication of OP might be the absence of improvement in clinical conditions despite treatment with empirical antibacterial, antiviral, or antifungal therapies [5]. The treatment of this lung disease depends on its severity, with mild cases often resolving without the need for specific therapy [6]. However, persistent and more severe cases may require treatment with steroids, which are gradually tapered over several months [6].

Major complications of SOP include bacterial superinfections and viral pneumonia, particularly in older adults and those with chronic conditions. While OP after influenza is rare, distinguishing it from bacterial superinfections is challenging, yet critical, as OP requires specific treatment. The incidence of subsequent pneumonia for influenza types A and B in hospitalized patients is 68.6% and 56.9%, respectively [7]. The use of antiviral agents alone has not been proven effective in preventing the development of SOP [8]. Early clinical diagnosis and steroid treatment are very effective; however, relapses can still occur once the treatment is discontinued [2].

## Case presentation

**Figure 1:**
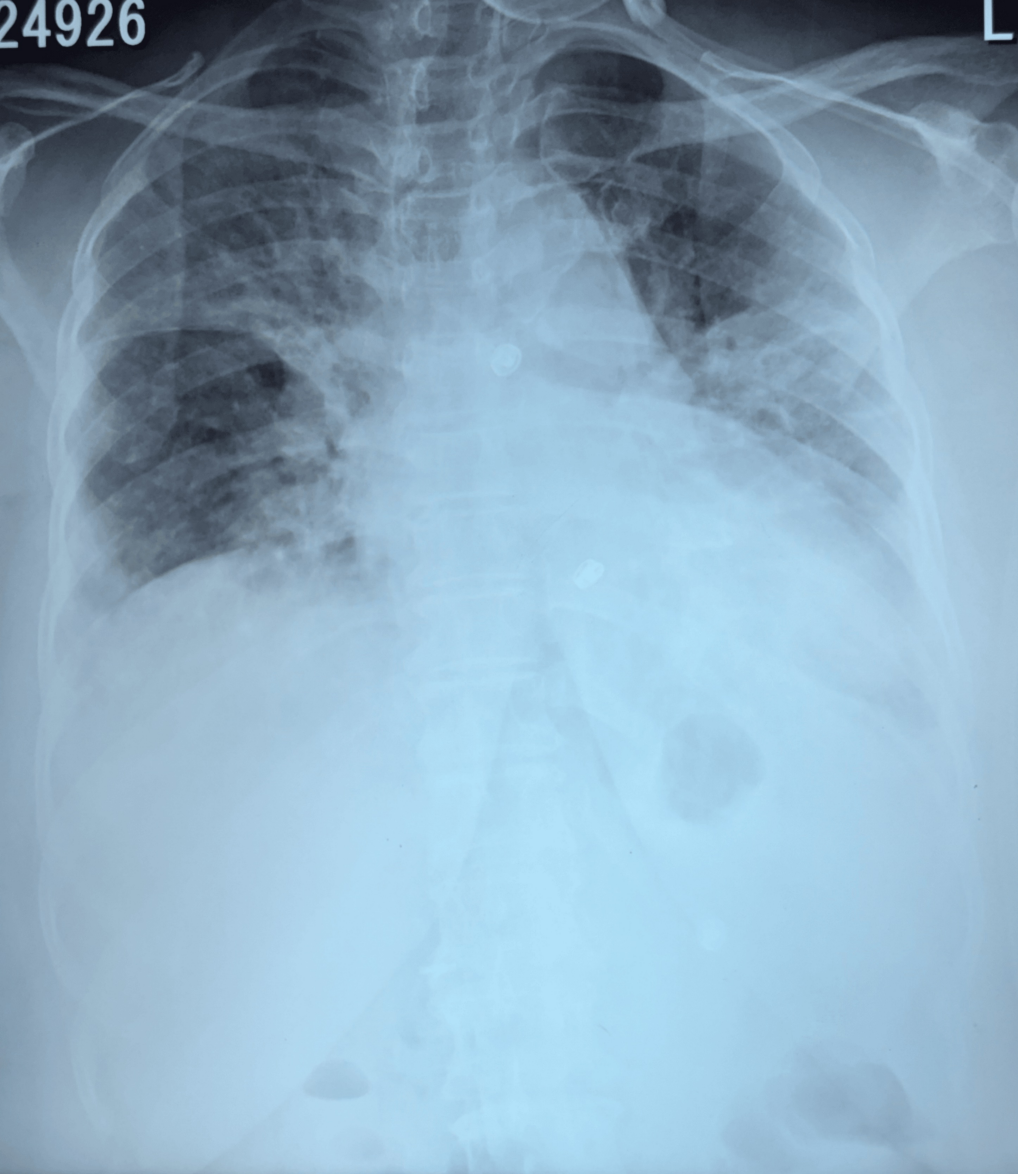
Chest X-ray showing bilateral infiltrates on admission

A 62-year-old woman with a medical history of hypertension and dyslipidemia was admitted to the medical ward with complaints of fever and productive cough for five days. She had no history of smoking. On admission, she was dyspnoeic with a respiratory rate of 20 breaths per minute; her saturation was 85% on room air, her pulse rate was 98 beats per minute, her blood pressure was 110/70 mmHg, and auscultation revealed bilateral diffused coarse crepitation. She was diagnosed with influenza A by nasopharyngeal antigen test reverse transcription-polymerase chain reaction (RT-PCR). Despite receiving antiviral therapy with oseltamivir 75 mg twice daily, her flu symptoms, including cough and fever, persisted. Also, she had an acute type 1 respiratory failure (pH: 7.42, pCO_2_: 35.3 mmHg, pO_2_: 44.3 mmHg) with elevation of inflammatory markers (CRP: 210 mg/L, ESR: 98 mm/h). Chest radiography (Figure 1) showed areas of consolidation and peripheral subpleural opacities.

**Figure 2:**
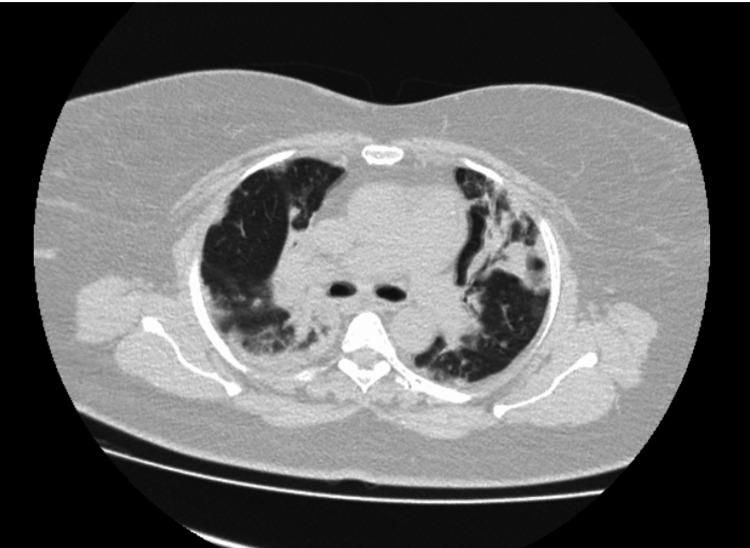
Chest CT showing bilateral consolidation on both lungs on admission CT: computed tomography

As per the microbiologist’s recommendation, broad-spectrum antimicrobial coverage with intravenous meropenem and teicoplanin was empirically administered alongside oseltamivir. No microorganisms were detected in the blood or sputum culture. High-resolution CT (HRCT) confirmed organizing pneumonia (Figure 2). She was ultimately diagnosed with SOP linked to influenza A. Initially she was treated with intravenous methylprednisolone 1 g daily for three days followed by oral prednisolone 1 mg/kg daily for two weeks, with significant improvement in chest radiography and inflammatory markers. Our medical team discharged her on day 18. The dose was gradually tapered off to 0.75 mg/kg, then to 0.5 mg/kg daily, and finally 0.25 mg/kg daily each month. With radiological and symptomatic improvement, the patient showed remarkable progress over three months. Table 1 presents a summary of the relevant investigations.

**Table 1:** Summary of investigations

Investigations				
Test	Patient value	Normal value		
White blood cells, x 10^3^/uL	12	4-11		
Hemoglobin, g/dL	15.4			11-15
Mean corpuscular volume, fL	84			80-100
Platelet count, x 10^3^/mm^3^	353			150-450
Blood picture	Neutrophil leukocytosis with left shift and few toxic granules			
Erythrocyte sedimentation rate, mm/hr	98			<10
C-reactive protein, mg/dL	210			<6
Estimated glomerular filtration rate, mL/kg/1.73m^2^	127			>90
Serum sodium, mmol/L	135			136-145
Serum potassium, mmol/L	3.6			3.5-5.1
Serum total calcium, mmol/L	2.24			2.12-2.62
Aspartate transaminase, U/L	32			15-37
Alanine transaminase, U/L	49			12-78
Serum bilirubin, mg/dL	0.7			0.2-0.8
Alkaline phosphatase, U/L	50			46-116
Serum albumin, g/L	42			34-50
Serum globulin, g/L	35			22-48
International normalized ratio	1.07			<1.2
Activated partial thromboplastin time, sec	31.5			27-42
Procalcitonin, ng/mL	0.7			>2.0 sepsis
Urine full report	Normal			
Fasting blood sugar, mg/dL	104			<126
HbA1c, %	5.4			<6
Transthoracic echocardiogram	Normal systolic and diastolic function			
Chest radiograph	Bilateral consolidation in the middle and lower lobes, consistent with bilateral pneumonia			
High-resolution computed tomography of the chest	Peripheral subpleural opacities and consolidations bilaterally with interseptal thickness			
Total cholesterol, mg/dL	188			<200
Triglycerides, mg/dL	89			<150
High-density lipoprotein cholesterol, mg/dL	46			50-59
Low-density lipoprotein cholesterol, mg/dL	124			<100
Very low-density lipoprotein cholesterol, mg/dL	18			2-30
Cholesterol-to-HDL ratio	4.1		<5:1	
Thyroid-stimulating hormone, uIU/mL	1.108		0.4-4	
TB polymerase chain reaction	Mycobacterium tuberculosis not detected			
Sputum culture	No growth			
Blood culture	No growth			

## Discussion

Post-influenza OP is an uncommon complication of influenza virus infection, with only a few cases reported in the literature. An accurate diagnosis is critical, as untreated SOP can be life-threatening. It is well known that SOP could occur in patients infected with H1N1 and avian flu viruses due to cytokine storm-mediated lung injury. Influenza viral pneumonia usually develops four to five days after the onset of illness, whereas OP may appear two to three weeks after the initial influenza symptoms [9,10]. In our case, the patient developed pneumonia five days after the onset of the illness. SOP should be suspected if cough, fever, and worsening difficulty breathing persist in influenza patients after initial improvement of influenza-like symptoms.

TBLB should be performed in these patients, as appropriate treatment for OP involves corticosteroid therapy. Due to the unavailability of a TBLB facility in our center, we performed HRCT-chest in our case. In general, OP following severe viral infections, including COVID-19, has shown a good response to corticosteroid treatment, except in a case of acute fibrinous OP (AFOP) caused by influenza A/H1N1 pneumonia after lung transplantation [11]. Our patient's condition improved remarkably after corticosteroid treatment. Many case reports suggest that SOP should be considered in the differential diagnosis when managing untreatable respiratory failure and that an open lung biopsy may be the most accurate method for diagnosing SOP [4]. As per recent literature, the causes of AFOP are either idiopathic or unknown, or secondary to infections, drug exposure (e.g., amiodarone, statins), and autoimmune collagen vascular diseases [11].

## Conclusions

Severe respiratory failure and SOP can occur following influenza A infection, even after the initial influenza symptoms have resolved. Clinical suspicion for SOP should be raised when a patient requires increasing oxygen levels and exhibits atypical clinical features despite receiving treatment for pneumonia or another underlying lung condition.
